# Comparison of RNA Marker Panels for Circulating Tumor Cells and Evaluation of Their Prognostic Relevance in Breast Cancer

**DOI:** 10.3390/cancers15041271

**Published:** 2023-02-16

**Authors:** Eva Welsch, Eva Schuster, Michael Krainer, Maximilian Marhold, Rupert Bartsch, Michael B. Fischer, Michael Hermann, Gabriele Hastermann, Heidemarie Uher, Gerhard Sliutz, Birgit Anker, Robert Zeillinger, Eva Obermayr

**Affiliations:** 1 Department of Obstetrics and Gynecology, Comprehensive Cancer Center, Medical University of Vienna, 1090 Vienna, Austria; 2 Department of Medicine I, Clinical Division of Oncology, Comprehensive Cancer Center, Medical University of Vienna, 1090 Vienna, Austria; 3 Department of Blood Group Serology and Transfusion Medicine, Medical University of Vienna, 1090 Vienna, Austria; 4 Center for Biomedical Technology, Department for Biomedical Research, Danube University Krems, 3500 Krems, Austria; 5 Brustgesundheitszentrum, Department of Surgery, Klinik Landstaße, Wiener Gesundheitsverbund, 1030 Vienna, Austria; 6 Brustgesundheitszentrum, Department of Gynecology and Obstetrics, Klinik Landstaße, Wiener Gesundheitsverbund, 1030 Vienna, Austria

**Keywords:** circulating tumor cells, qPCR, gene expression, liquid biopsy, tumor marker panel

## Abstract

**Simple Summary:**

Circulating tumor cells are precursors of distant metastasis in many cancer types. These cells circulate in the peripheral blood, which can be analyzed in a non-invasive procedure to identify patients at risk to develop metastases. In order to improve non-invasive diagnostic procedures for breast cancer, we aimed at investigating gene transcripts as liquid biopsy markers in the blood employing density gradient centrifugation as a cost- and time-saving method for the isolation of the target cells. We were able to detect the selected biomarkers in 86.1% of metastatic and 31.1% of early breast cancer patients. The presence of some markers were significantly related with shorter survival of the patients. Our data suggest that these transcripts have the potential to identify patients with poor prognosis who might benefit from further clinical intervention.

**Abstract:**

Liquid biopsy is a promising tool for therapy monitoring of cancer patients, but a need for further research in this field exists in order to improve sensitivity, specificity, standardization and minimize costs. In our present study, we evaluated two panels of transcripts related with the presence of circulating tumor cells (CTCs) (Panel 1: *CK19*, *EpCAM*, *SCGB2A2* and Panel 2: *EMP2*, *SLC6A8*, *HJURP*, *MAL2*, *PPIC* and *CCNE2*) in two cohorts of breast cancer patients (metastatic and early). A blood cell fraction possibly containing CTCs was isolated with density gradient centrifugation, followed by RNA isolation and qPCR using TaqMan^®^ or RT-qPCR using hybridization probes. The positivity rates of the investigated panels were similar, albeit higher in metastatic (69.4% Panel 1, 75.0% Panel 2; total 86.1%) compared to early (18.9% Panel 1, 23.3% Panel 2; total 31.1%) breast cancer patients. *CK19*, *SCGB2A2*, *EMP2*, *HJURP*, *MAL2*, and *CCNE2* individually correlated with shorter overall survival in the metastatic patient cohort. The findings highlight the additional value of Panel 2 markers, which are in contrast to *CK19* and *EpCAM* not solely linked to an epithelial phenotype.

## 1. Introduction

Breast cancer is one of the most common cancers in women worldwide [1]. Depending on the biological subtype of the tumor defined by HER2 staining pattern or gene amplification, or on patient characteristics such as tumor size, lymph node involvement, and disease stage at first diagnosis, therapy options and patient outcomes vary. In more than 95% of the cases, breast cancer is detected at an early stage, resulting in favorable prognosis. In the metastatic stage, however, where the treatment focuses on prolonging patients survival rather than cure [2], patients exhibit much less favorable prognosis. As tumor recurrence or distant metastasis can occur more than 20 years after primary diagnosis [3] (especially in the hormone receptor-positive setting), early detection of micrometastasis by circulating tumor cell (CTC) analysis through liquid biopsy screenings can help predict patient prognosis and could improve individual treatment [4]. So far, numerous studies have shown the clinical relevance of CTC detection in breast cancer, including its association with poor overall, disease-free and distant-metastasis-free survival [5,6,7,8,9,10].

Since the discovery of CTCs more than a century ago, only two technologies have been cleared by the Food and Drug Administration (FDA) for clinical application in metastatic breast cancer: the CellSearch system, based on the immunomagnetic enrichment of epithelial CTCs (Menarini Silicon Biosystems, Huntingdon Valley, PA, USA) [11], and the Parsortix^®^ system (Angle plc., Guildford, UK), a label-free microfluidic separation based on cell size and deformability [12]. Nevertheless, miscellaneous CTC isolation methods with different principles have been developed, based on physical (size, density, deformability, bioelectricity) or functional properties (adhesion, protein secretion) of the cells of interest, such as filtration, density gradient centrifugation, and immunocapture (positive or negative selection) or combined methods [13]. Every CTC isolation method has its pros and cons; for example, density gradient centrifugation is characterized by a high yield and by enrichment of CTCs independent of their phenotype at the cost of a high number of residual blood cells in the enriched sample [14]. Therefore, it is important to choose the right method based on the research question asked.

We previously established a panel of six gene transcripts possibly related to the presence of CTCs using OncoQuick (Greiner Bio-One, Kremsmünster, Austria) density gradient centrifugation. The six-gene panel was superior to the commonly used marker genes for epithelial breast cancer CTCs, *EpCAM* and human mammaglobin A (*SCGB2A2*), both in samples from patients with early and advanced breast cancer [15].

In the present study, we evaluated the six-gene panel in a larger cohort of patients with early and metastatic breast cancer in order to investigate the panels’ clinical potential, and added the analysis of *CK19*, a highly specific marker for epithelial CTCs [16]. Thus, we assigned *SCGB2A2*, *CK19* and *EpCAM* to Panel 1, and the previously identified six genes *MAL2*, *PPIC*, *CCNE2*, *HJURP*, *EMP2* and *SLC6A8* to Panel 2. We compared the positivity rates of both panels in the patients’ samples after a density gradient based enrichment of a blood cell fraction possibly containing CTCs. The larger cohort of patients compared to our previous study further allowed us to evaluate the prognostic significance of the included gene markers. Our data suggest that Panel 2 markers can have an additive value to the detection of epithelial markers alone, and that they may even have the potential to identify patients with poor prognosis who might benefit from further clinical intervention.

## 2. Materials and Methods

### 2.1. Patients and Blood Samples

The study included 36 patients with metastatic breast cancer recruited at the Division of Oncology, Department of Medicine I, Vienna General Hospital, Austria and 90 patients with early breast cancer recruited at the Brustgesundheitszentrum Klinik Landstraße Vienna, Austria. The latter were admitted for undergoing a tissue biopsy after a suspicious mammography finding, and the diagnosis was confirmed by histopathologic analysis of tissue specimen. All patient blood samples were drawn aseptically prior to treatment and after discarding the first 1–5 mL of blood, which might contain contaminating epithelial skin cells. From the patients with metastatic breast cancer, the peripheral blood was drawn in two 9 ml Vacuette K_3_EDTA tubes (Greiner Bio-One, Kremsmünster, Austria). From the early breast cancer patients the blood was drawn in three CPT™ blood collection tubes (Becton Dickinson, Franklin Lakes, NJ, USA). After blood draw, the CPT™ tubes were inverted several times and immediately shipped at ambient temperature with a courier to the Medical University of Vienna for further processing. Additionally, 28 female healthy blood donors (HD) without any known history of cancer were recruited as control group at the Department of Blood Group Serology and Transfusion Medicine, Medical University of Vienna, Vienna, Austria. The blood samples from these HD were taken either in Vacuette K_3_EDTA tubes (n = 9) or in CPT™ tubes (n = 19) in order to match to the respective group of patients. All patients and donors gave their written informed consent for the analysis of their specimen. This study was approved by the Ethics Committee of the Medical University of Vienna, Austria (EK366/2003 and EK2266/2018).

### 2.2. Processing of Blood Samples from Metastatic Breast Cancer

The blood mononuclear cell (PBMC) fraction possibly containing CTCs was isolated on the same day by density gradient centrifugation using Histopaque 1077 (HP) (Sigma, St. Louis, MO, USA). In short, the contents of the two Vacuette K_3_EDTA tubes were combined and filled up to a total volume of 30 mL with PBS. Then, each 10 mL of diluted blood was gently added on top of 5 mL HP density gradient medium in a 15 mL tube. After centrifugation at 400× *g* for 30 min with inactivated breaks, the PBMCs in the opaque interface were aspirated, washed with PBS and centrifuged again at 250× *g* for 10 min. The supernatant was discarded, and the PBMC pellets lysed with RLT RNA lysis buffer (Qiagen, Hilden, Germany) containing 1% β-Mercaptoethanol. Blood samples taken in Vacuette K_3_EDTA tubes from the HD group were processed alike. Cell lysates were kept at −80 °C until RNA extraction.

### 2.3. Processing of Blood Samples from Early Breast Cancer

The CPT™ tubes were centrifuged at 1600× *g* for 20 min. The upper plasma layer was discarded, the opaque layers containing PBMCs were combined in a new tube and filled up to a total of 50 mL with PBS, mixed and centrifuged at 300× *g* for 15 min. The supernatant was discarded, the PBMC pellet washed in PBS and centrifuged again at 300× *g* for 10 min. The cell pellet was lysed in RLT RNA lysis buffer (Qiagen, Hilden, Germany) containing 1% β-Mercaptoethanol. Blood samples taken in CPT™ tubes from the HD group were processed alike. Cell lysates were kept at −80° C until RNA extraction.

### 2.4. Spiking Experiment Histopaque

The breast cancer cell lines MCF-7, Hs578T and SKBR-3 (obtained from the American Type Culture Collection; ATCC, Manassas, VA, USA) were grown in RPMI-1640 medium, supplemented with 10% fetal bovine serum and 1% penicillin-streptomycin (Invitrogen, Waltham, MA, USA) at 37 °C, 5% CO_2_. The cell lines were tested negative for mycoplasma contamination. At about 70% confluence, the tumor cells were trypsinized and counted with the LUNA cell counter (Logos Biosystems, Anyang-si, South Korea). Each 10, 100, and 1000 tumor cells were spiked in duplicates into 5 mL peripheral blood of a HD. A blood sample without the addition of tumor cells was also included in duplicates. The samples containing the tumor cells and the unspiked control sample were processed by density gradient centrifugation using Histopaque 1077 as described above. Cell pellets were lysed in RLT RNA lysis buffer (Qiagen, Hilden, Germany) containing 1% β-Mercaptoethanol. Cell lysates were kept at −80 °C until RNA extraction.

### 2.5. mRNA Isolation

After thawing the cell lysates obtained from patient blood samples, HD, and from the spiking experiment, the total RNA was isolated utilizing the RNeasy Mini Kit (Qiagen, Hilden, Germany) without DNase treatment. The RNA concentration was measured using the QuantiFluor RNA System (Promega, Madison, WI, USA) on the Qubit fluorometer (ThermoFisher, Waltham, MA, USA). Then, the mRNA was isolated from the total RNA with the Dynabeads mRNA purification kit (ThermoFisher, Waltham, MA, USA). The isolation was carried out according to the manufacturer’s instruction. Per 5 µg total RNA, 13 µL of magnetic bead suspension was used. The captured mRNA was eluted in 20 µL 10 mM Tris-HCl pH 7.5. The mRNA was then split into two equal halves for subsequent TaqMan^®^-based qPCR and RT-qPCR using FRET probes, respectively.

### 2.6. Preamplification and TaqMan^®^-Based qPCR

Half of the yielded mRNA was reverse transcribed into cDNA using the SuperScript VILO Mastermix (Invitrogen, Waltham, MA, USA) (25 °C for 10 min, 42 °C for 1 h, 85 °C for 5 min, 4 °C). The targets of interest (*EpCAM*, *CCNE2*, *PPIC*, *MAL2*, *EMP2*, *HJURP*, *SCGB2A2* and *SLC6A8*) as well as the reference gene *CDKN1B* were quantified using the TaqMan^®^ Universal Mastermix II (ThermoFisher, Waltham, MA, USA) and exon spanning TaqMan^®^ assays (ThermoFisher, Waltham, MA, USA) in duplicates after a target-specific pre-amplification step. The Pre-amplification was carried out on the 2720 Thermal Cycler (Applied Biosystems, Waltham, MA, USA) (25 °C for 5 min, 95 °C for 10 min, 10 cycles with 95 °C for 15 s and 60 °C for 4 min). The qPCR was carried out on the QuantStudio™ 7 Flex Real-Time PCR System (ThermoFisher, Waltham, MA, USA) with standard thermal cycling conditions (50 °C for 2 min, 95 °C for 10 min, 40 cycles of 95 °C for 15 s and 60 °C for 1 min). Raw data were analyzed using the QuantStudio Software (ThermoFisher, Waltham, MA, USA) (v1.6.1) with automatic threshold setting and baseline correction.

### 2.7. CK19 RT-qPCR Using FRET Probes

The remaining half of the mRNA volume was used to determine *CK19* gene expression using FRET probes and primers hybridizing to the CK19 gene [16]. Two different types of RT-qPCR mastermixes were used: (i) For the early breast cancer samples and corresponding HD samples, the reaction was carried out using the Luna^®^ Universal Probe One-Step RT-qPCR Kit at the LightCycler 480 II (Roche, Basel, Switzerland) (55 °C for 10 min, 95 °C for 1 min, followed by 50 cycles of 95 °C for 10 s and 63 °C for 30 s and one cooling cycle with 40 °C for 30 s); (ii) for the metastatic breast cancer samples and corresponding HD controls the commercially available CK-19 Kit (OncoLab Diagnostics, Wiener Neustadt, Austria) was used.

### 2.8. Calculation of Cut-Off Threshold Values

If a marker was detected in the HD samples, a threshold was set allowing a maximum of 10% positive findings in the HD group. This threshold value was calculated for each transcript by subtracting an integer multiple of the standard deviation from the mean Ct value of the HD samples. If a marker was not detected in the HD samples, a Ct value of 35 (45 for *CK19*) was defined as a threshold. Every patient sample with a mean Ct value below the threshold was defined as positive for the respective marker. If only one technical replicate was detected, the sample was defined as negative for that marker. The overall positivity of a panel was defined as positivity of at least one of the markers assigned to the respective panel (Panel 1: *CK19*, *EpCAM*, *SCGB2A2*; Panel 2: *EMP2*, *SLC6A8*, *HJURP*, *MAL2*, *PPIC*, *CCNE2*).

### 2.9. Statistics

Fisher’s exact test and Chi-Square test were used to compare the marker positivity between the patient with metastatic and early breast cancer and associations with clinical parameters. McNemar test was used to compare the positivity of Panel 1 and Panel 2 in each individual cohort. Kaplan-Meier analysis and log rank test was used to compare the overall survival of patients with marker positivity or negativity. Overall survival (OS) was defined as time between blood collection and the event of death or the last observation date of the patient. For each marker within a panel, the log-rank *p*-values were adjusted for multiple testing using Bonferroni correction (R package survival: 3.4–0). Two-sided tests were used at all analyses. GraphPad PRISM (version: 8.4.3) and R (version: 4.2.2) were used for graphics design and analysis.

## 3. Results

### 3.1. Spiking Experiment Histopaque Density Gradient Centrifugation

In a spiking experiment with 10, 100 and 1000 tumor cells of three breast cancer cell lines with different subtypes (SKBR3 hormone receptor (HR)-negative/HER2-positive, MCF-7 HR-positive/HER2-positive, Hs578T HR-negative/HER2-negative) into HD blood, the efficiency of the individual markers in combination with the CTC isolation with Histopaque density gradient centrifugation was evaluated (Figure S1). The detection of as few as 10 SKBR3 cancer cells isolated from a peripheral blood matrix (5 mL) was possible using the markers *CK19*, *SCGB2A2*, *EpCAM*, *EMP2*, *MAL2* and *PPIC* (Figure S1a). *CK19* and *SCGB2A2* were not detectable in HD PBMCs at all. *EpCAM*, *EMP2*, *MAL2*, and *PPIC* show background gene expression levels in the unspiked blood sample; however, the steadily increasing gene expressions relative to the unspiked sample with increasing numbers of spiked SKBR3 tumor cells indicate that these markers may be appropriate for the detection of CTCs as well (Figure S1a). In the MCF-7 and Hs578T cell lines lacking *EpCAM*, *SCGB2A2* or *CK19*, the presence of *EMP2*, *MAL2* or *PPIC* transcripts was still observed in the spiked samples, albeit at lower levels than in SKBR3 (Figure S1b,c). Using the other markers (*SLC6A8*, *HJURP*, *CCNE2*), the detection of the spiked-in tumor cells was not possible due to high background signal from PBMCs and the low expression in the selected three cell lines, which results in equal Ct values in spiked and unspiked samples.

### 3.2. Evaluation of Gene Panels in Metastatic Breast Cancer Patients

The CTC/PBMC fractions of metastatic breast cancer patients and HD were isolated with Histopaque density gradient centrifugation. The expression of all markers was quantified from the isolated mRNA using TaqMan^®^ probe qPCR and hybridization probe RT-qPCR for *CK19*, respectively. Due to background expression of all markers but *SCGB2A2* and *CK19* in the HD group, a threshold level was calculated for these markers to define positive patient samples.

Both panels had similar detection rates (Panel 1 69.4%, Panel 2 75.0%; McNemar test *p* = 0.754; Table 1). From the 31 patients positive with any of the markers, 21 (67.7%) were positive with both panels, four (12.9%) with Panel 1 only, and six (19.4%) with Panel 2 only.

**Figure 1:**
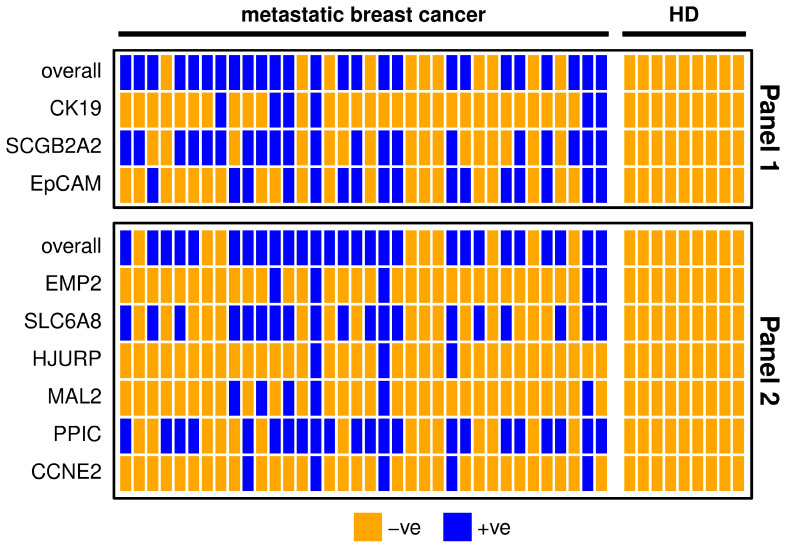
Heatmap showing metastatic breast cancer patient (n = 36) and healthy donor (HD, n = 9) samples for each marker separately and overall. For the definition of positivity, a threshold was set considering the gene expression levels in the HD group, in order to allow a maximum of 10% positive findings in the HD group. Samples with positive findings are marked blue and with negative findings orange. Panel 1 is shown in the upper graph and Panel 2 in the lower graph.

The markers *SCGB2A2*, *EpCAM*, *SLC6A8* and *PPIC* were detectable in about half of the metastatic breast cancer samples. In contrast, *CK19*, *EMP2*, *HJURP*, *MAL2* and *CCNE2* were detected in less than one third of the metastatic breast cancer patients (Table 1). The results of both marker panels coincide in about 70% of metastatic breast patient samples (Figure 1). Five samples are negative for all of the included gene transcripts, which might indicate that no CTCs or only CTCs are present that are negative for the investigated markers. The possibility of poor RNA yield could be ruled out due to the conformation of sufficient RNA quantity by the expression analysis of the reference gene *CDKN1B*. One patient sample was positive for all investigated markers, which might indicate a high number of CTCs in the blood. In Panel 1, the two markers with the highest positivity rate (*SCGB2A2* and *EpCAM*) may have additive value for CTC detection; *CK19* however does not detect additional positives (Figure 1). Both *CK19* and *SCGB2A2* were not expressed in any HD samples. Nevertheless, after applying the calculated threshold value, none of the HD samples remained positive for any other marker as well. In Panel 2, the markers with the lower positivity rates (*EMP2*, *HJURP*, *MAL2*, and *CCNE2*) do not detect additional positives to the markers with higher positivity rates (*SLC6A8*, *PPIC*). Combining the two panels resulted in an overall positivity rate of 86.1% achieved by the markers *SCGB2A2*, *EpCAM*, *SCL6A8* and *PPIC* alone.

### 3.3. Evaluation of Gene Panels in Early Breast Cancer Patients

Early breast cancer patients’ and HD’ CTC/PBMC fractions were isolated by density gradient centrifugation using the CPT™ system. The expression of all markers was assessed using TaqMan^®^ probes and hybridization probes for *CK19*, respectively. Due to background expression of all markers but *SCGB2A2* in the HD group, a threshold level was calculated for these markers to define positive patient samples.

The positivity rate was significantly lower (Chi-Square-test *p* < 0.001) in the early (18.9% positivity with Panel 1, 23.3% with Panel 2) (Table 2) compared to the metastatic patient samples (69.4% positivity with Panel 1, 75.0% with Panel 2) (Table 1). In the early breast cancer cohort, both panels had similar detection rates (McNemar test *p* = 0.481; Table 2). From the 28 patients positive with any of the markers, ten (35.7%) were positive with both panels, seven (25.0%) with Panel 1 only, and eleven (39.3%) with Panel 2 only.

**Figure 2:**
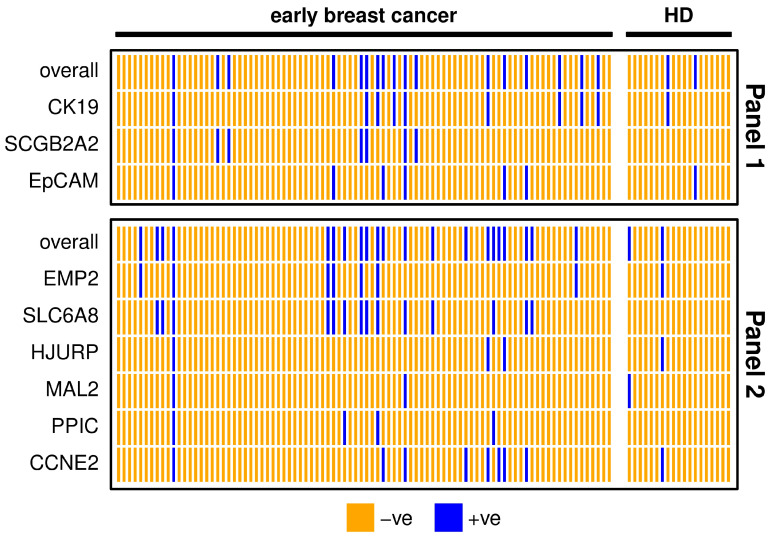
Heatmap showing positive (blue) and negative (orange) early breast cancer patients’ (n = 90) and healthy donor (HD, n = 19) samples for each marker or overall, comparing markers from Panel 1 (upper graph) and Panel 2 (lower graph). For the definition of positivity, a threshold was set considering the gene expression levels in the HD group, in order to allow a maximum of 10% positive findings in the HD group.

The detection rate of both marker panels in early breast cancer patients was about double as the positivity rate in HD samples (Table 2), resulting from calculated thresholds. The three markers *SCGB2A2*, *SLC6A8* and *PPIC* were defined negative in all HD samples after applying the threshold and *SLC6A8* had the highest positivity rate. The two marker panels coincided in 80% of early breast cancer patient samples (Figure 2). One patient was positive for all of the investigated markers, indicating a high chance of CTC presence in the blood. All individual markers from Panel 1 contribute to the overall positivity of the panel. *MAL2* and *PPIC* did not detect additional positives to the other markers included in Panel 2.

### 3.4. Association of CTC-Related Markers with Clinical Characteristics of the Patients

The association of the markers’ positivity and clinical characteristics of the metastatic breast cancer cohort including histopathological subtype of the tumor, metastasis in bone, liver, lung or brain, number of metastasis sites and lines of previous treatment was assessed using Fisher’s exact test and Chi-Square test. We observed a higher rate of *EpCAM* positivity in patients who had already received multiple lines of treatments, and a lower rate of *SLC6A8* positive patients with lung metastasis; however, after adjusting the *p*-values for multiple testing none of these associations remained significant. No other association between marker positivity and clinical features could be observed (Table S1). Among the eight hormone receptor negative patients, four (50.0%) were solely positive for Panel 2, whereas from the 22 hormone receptor positive patients just two patients (9.1%) were Panel 2 positive (Fisher’s exact test *p* = 0.029).

Among the early breast cancer patients, the marker expression was compared to clinical parameters such as patient age, HER2 status, estrogen (ER) and progesterone receptor (PR) status, lymphangioinvasion, KI 67 label index, and tumor grade. Positivity of Panel 1 was significantly higher in patients with high-grade tumors (Table S2). None of the individual markers was associated with any of the clinical parameter. Nevertheless, we observed that among the positive early breast cancer patients, the patients only positive for Panel 2 were significantly more likely HER2-positive. From the patients with known HER2 status, five of the seven HER2-positive patients (71.4%) were solely positive for Panel 2, whereas from the 12 HER2-negative patients just a single patient (8.5%) was Panel 2 positive (Fisher’s exact test *p* = 0.010). The early breast cancer patients were observed for a median follow-up period of 26.8 months, and within this time, 7/90 (7.7%) patients relapsed. Due to the short follow-up, we did not perform a survival analysis in this patient cohort. Clinical and pathological characteristics of patients are shown in Tables S1 and S2.

### 3.5. Marker Expression and Overall Survival of Metastatic Breast Cancer Patients

**Figure 3:**
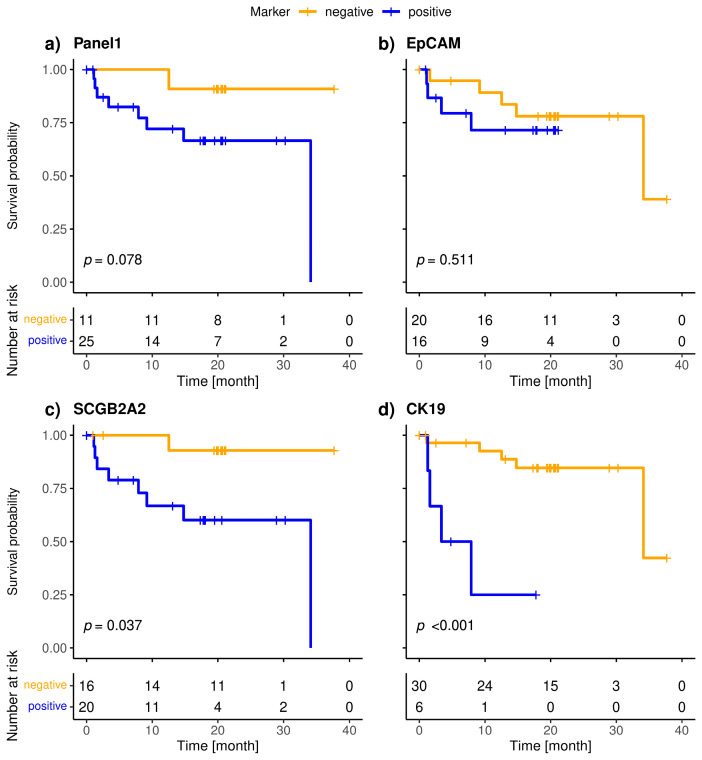
Kaplan-Meier plots of overall survival of metastatic breast cancer patients stratified by positivity (blue) of Panel 1 (**a**), *CK19* (**b**), *SCGB2A2* (**c**) and *EpCAM* (**d**); Bonferroni corrected *p*-values are shown, *p* < 0.05 is defined as level of significance.

**Figure 4:**
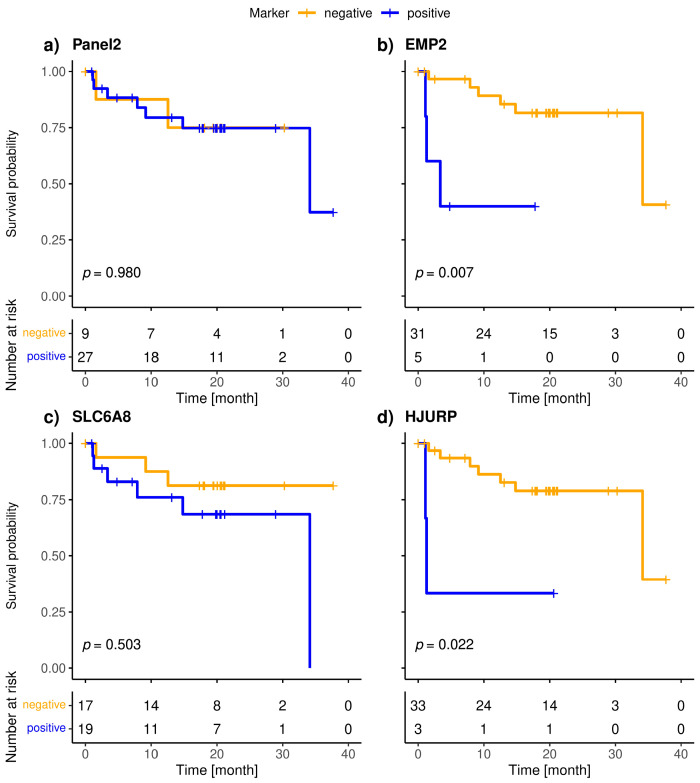

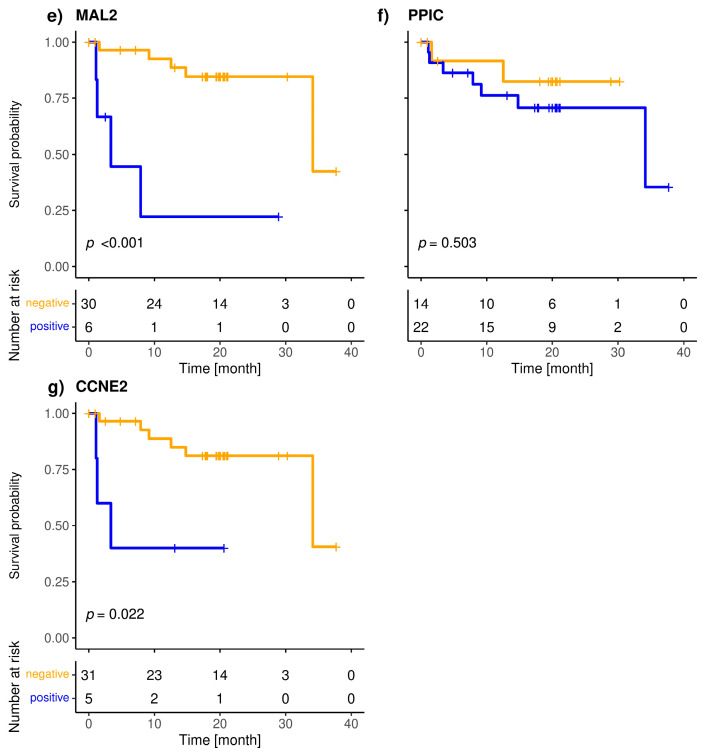
Kaplan-Meier plots of overall survival of Panel 2 (**a**), *EMP2* (**b**), *SLC6A8* (**c**), *HJURP* (**d**), *MAL2* (**e**), *PPIC* (**f**) and *CCNE2* (**g**) positive (blue), and negative (orange) metastatic breast cancer patients; Bonferroni corrected *p*-values are shown, *p* < 0.05 is defined as significant.

The included patients exhibited a median overall survival of 34.1 months, and 9/36 (25.0%) of the patients died during the observation period. The median follow-up of the patients was 20 months. The highly specific epithelial (*CK19*) and breast cancer specific (*SCGB2A2*) marker were significantly associated with shorter overall survival (Figure 3). The median OS of *CK19*-positive patients was 5.6 months, whereas it was 34.1 months for the *SCGB2A2*-positive patients. Among the Panel 2 markers contributing to positive findings in fewer patients, *EMP2, HJURP, MAL2*, and *CCNE2* were associated with short OS (Figure 4); in contrast, *EpCAM*, *SLC6A8* and *PPIC* were not related with OS. The six markers with prognostic relevance (*CK19*, *SCGB2A2*, *EMP2*, *HJURP*, *MAL2*, and *CCNE2*) detected in total 58.3% of metastatic breast cancer patients.

## 4. Discussion

We evaluated a previously identified panel of six gene transcripts related to the presence of CTCs in patients in a larger cohort of early and metastatic breast cancer in order to investigate its clinical importance, and as in our previous study, we included *EpCAM* and *SCGB2A2* as common markers for epithelial and breast cancer cells. In addition we assessed *CK19* employing a highly specific assay [16], which does not require a high depletion of unwanted blood cells prior to the analysis. For this reason, we performed an enrichment using Histopaque 1077 (HP) density gradient medium or CPT™ blood collection tubes, which provide a single-step standardized method for the isolation of PBMCs and possibly CTCs. However, it remained yet to evaluate whether the analysis of other CTC-related gene transcripts, as our previously identified gene panel, would be feasible as well.

Using HP density gradient centrifugation to isolate PBMCs and possibly CTCs from metastatic breast cancer patients’ blood, we were able to achieve an overall positivity of 86.1%. The investigated 6-gene marker panel (Panel 2) was positive in 75.0% of metastatic patients, compared to the epithelial markers *CK19*, *EpCAM* and *SCGB2A2* (Panel 1), which were positive in 69.4% cases. In our previous pilot study on the identification of novel markers, we were able to reach a similar positivity, detecting transcripts in 80.6% of metastatic breast cancer patients using the same 6-gene panel [15]. Other studies employing different CTC enrichment systems detected less positive metastatic breast cancer patients. Using the Parsortix^®^ enrichment in combination with qPCR analysis of the markers (Panel 2) we detected the same transcripts in 53% metastatic and 23% early breast cancer patient samples [17]. The DETECT study reached a 50% positivity using the CellSearch system and just 40% with the AdnaTest Breast [18]. Cohen et al. detected CTCs in 48.5% metastatic breast cancer patients using the Parsortix^®^ PC1 System and cytological evaluation of the harvested cells [19]. Differences in detection rates might be explained by the CTC isolation techniques and the CTC population isolated based on the characteristics of the method.

Additionally, we were able to detect CTC-related transcripts in 31.1% CPT™-enriched blood samples of early breast cancer patients. Our positivity rate of early breast cancer patient samples is in line with others reporting 9% to 55% positive patients [20]. The lower rate of positive samples among the early breast cancer patients compared to metastatic breast cancer patients can be explained by the lower numbers or even absence of CTCs thereby resulting in difficulties to isolate them. In principle, our spiking experiments demonstrated a high sensitivity of the overall approach detecting up to 10 tumor cells per 5 mL of HD blood. However, the expression of the selected markers can vary in breast cancer cell lines of different subtypes (as shown in Figure S1) and in CTCs.

Panel 2 emerged from our early study, in which we identified novel markers for the detection of CTCs using microarray analysis of 40 established cancer cell lines and healthy PBMCs [15]. The selection criterion was differential gene expression in the majority of the cell lines irrespective of their molecular subtype; thus, epithelial markers such as *EpCAM* or *SCGB2A2* had not been selected a priori in that study. For this reason, Panel 2 can be valuable when Panel 1 is not detected. Indeed, *EMP2* and *PPIC* transcripts in spiked blood samples indicated the presence of Hs579T breast cancer cells; in contrast, the analysis of the epithelial markers *EpCAM*, *CK19* and *SCGB2A2* alone would not have detected these hormone receptor and HER2-negative cells (Figure S1c). Overall, we did not observe major differences of the two investigated marker panels regarding positivity rates in the patient blood samples (metastatic: 69.4% and 75.0%, early: 18.9% and 23.3%, using Panel 1 and Panel 2, respectively). Future research may explore the question as to the detection of Panel 2 markers in the blood of hormone receptor negative patients.

The present study is the first to demonstrate the prognostic relevance of *MAL2*, *EMP2*, *CCNE2* and *HJURP* transcripts in liquid biopsy samples. *PPIC* detected in follow-up blood samples from ovarian cancer patients was shown to be associated with PFS [21]. Among the CTC-related transcripts in Panel 2, *SLC6A8* and *PPIC* had the highest positivity rate among the metastatic breast cancer patients; however, the presence of these markers beyond the calculated threshold was not associated with OS. In contrast, *MAL2*, *EMP2*, *CCNE2* and *HJURP* were positive in fewer cases but associated with shorter OS.

Different studies confirmed the CTC count as a prognostic factor for primary breast cancer, including its relevance for overall and disease free survival [22,23,24,25,26,27]. Quite recently Matikas et al. found a significant association of *CK19* mRNA positive CTCs and 10-year overall survival, disease-free survival, as well as correlations with larger tumor size and positive lymph nodes in a cohort of 1220 early and locally advanced breast cancer patients by using a comparable *CK19* primer/probe system as in the present study [5]. We were not able to observe an association of the investigated CTC-related transcripts with survival in early breast cancer, which might be explained by the short time of observation, as recurrence can occur 20 years or more after first appearance [28].

The *CK19* assay employing a sensitive and specific primer/probe system was designed not to amplify genomic DNA as well as known pseudogenes [16]. Due to the high specificity of the commercially available test (CK-19 Test, OncoLab Diagnostics, Wiener Neustadt, Austria), it can be assumed that samples being positive for this assay might be true positives and contain CTC. The worse OS of *CK19* positive metastatic breast cancer patients’ in our study may further support this assumption. In early breast cancer patients, we analyzed *CK19* expression using another RT-qPCR Mastermix, which also amplified some HD samples, compared to the metastatic cohort, where the transcript was not detected in any HD. This phenomenon may be explained by other influencing factors, for example buffer composition of RT-qPCR reagents.

Limitations of this study are the short follow-up of the early breast cancer patients, the small cohort size of metastatic breast cancer patients and the lack of age-matched controls, which may have confounded our results. Therefore, future studies with larger cohort sizes and a larger proportion of hormone receptor negative patients will be necessary to evaluate the efficacy of the markers.

Another point is the use of different protocols for the isolation of CTCs from metastatic and early patients. In order to reduce cost per sample, we used the HP gradient for in-house delivered patients’ samples (metastatic breast cancer) and switched to CPT™ tubes for samples with longer shipping (early breast cancer). The two used methods are highly comparable, because they are both based on the same density gradient medium. An advantage of the CPT™ system is that the blood is drawn directly into the blood collection tubes that contains the density medium and a physical barrier, which ensures that the separation of the enriched cells is maintained after centrifugation. Thereby we were able to reduce handling procedure time, because it is not necessary to dilute the blood and layer it onto a density gradient medium. Our analysis found that despite of the high background of contaminating leukocytes in the enriched samples, the detection of *CK19* and *SCGB2A2* was highly specific, and that the CTC-related markers assigned to Panel 2, which may not only be linked to an epithelial phenotype can be detected as well. The associations of the Panel 2 markers *MAL2*, *EMP2*, *CCNE2* and *HJURP* with short OS may point to different biological characteristics of the enriched cells and warrant further studies in aggressive breast cancer subtypes.

## 5. Conclusions

We evaluated two panels of transcripts related with the presence of CTCs in two cohorts of breast cancer patients. The positivity rates of the investigated panels were similar, albeit higher in metastatic compared to early breast cancer patients. The epithelial marker *CK19* and the breast cancer related marker *SCGB2A2* were significantly associated with shorter OS in the metastatic patients. The Panel 2 markers *EMP2, HJURP, MAL2*, and *CCNE2* individually correlated with shorter OS in the metastatic patient cohort. The findings highlight the additional value of Panel 2 markers, which are in contrast to *CK19* and *EpCAM* not solely linked to an epithelial phenotype. Our data suggest that these transcripts have the potential to identify patients with poor prognosis who might benefit from further clinical intervention.

## Figures and Tables

**Table 1:** Positivity rate of the CTC-related gene markers in metastatic breast cancer patients (n = 36) and HD (n = 9) after applying a cut-off threshold value. The positivity rates are given for each marker individually and for overall Panel 1 and Panel 2. For the definition of positive patient sample, a threshold was set in order to allow a maximum of 10% positive findings in the HD group.

Panel 1	Metastatic Breast Cancer	HD	Panel 2	Metastatic Breast Cancer	HD
overall	25 (69.4%)	0 (0.0%)	overall	27 (75.0%)	0 (0.0%)
*CK19*	6 (16.7%)	0 (0.0%)	*EMP2*	5 (13.9%)	0 (0.0%)
*SCGB2A2*	20 (55.6%)	0 (0.0%)	*SLC6A8*	19 (52.8%)	0 (0.0%)
*EPCAM*	16 (44.4%)	0 (0.0%)	*HJURP*	3 (8.3%)	0 (0.0%)
			*MAL2*	6 (16.7%)	0 (0.0%)
			*PPIC*	22 (61.1%)	0 (0.0%)
			*CCNE2*	5 (13.9%)	0 (0.0%)

**Table 2:** Positivity rate of the CTC-related gene markers in early breast cancer patients (n = 90) and healthy donors (HD, n = 19) after applying a cut-off threshold value. The positivity rates are given for each marker individually and overall for Panel 1 and Panel 2. For the definition of a positive patient sample, a threshold was set in order to allow a maximum of 10% positive findings in the HD group.

Panel 1	Early Breast Cancer	HD	Panel 2	Early Breast Cancer	HD
overall	17 (18.9%)	2 (10.5%)	overall	21 (23.3%)	2 (10.5%)
*CK19*	9 (10.0%)	1 (5.3%)	*EMP2*	7 (7.8%)	1 (5.3%)
*SCGB2A2*	7 (7.8%)	0 (0.0%)	*SLC6A8*	14 (15.6%)	0 (0.0%)
*EPCAM*	6 (6.7%)	1 (5.3%)	*HJURP*	3 (3.3%)	1 (5.3%)
			*MAL2*	2 (2.2%)	1 (5.3%)
			*PPIC*	4 (4.4%)	0 (0.0%)
			*CCNE2*	8 (8.9%)	1 (5.3%)

## Data Availability

The data presented in this study are available on request from the corresponding author.

## Supplementary Materials

The following supporting information can be downloaded at: https://www.mdpi.com/article/10.3390/cancers15041271/s1, Figure S1: Results of spiking experiment with (a) SKBR3, (b) MCF7 and (c) Hs578T breast cancer cells and analysis of marker expression with qPCR.; Table S1: Characteristics of the 36 patients with metastatic breast cancer and positivity rates of each marker and panels.; Table S2 Characteristics of the 90 patients with early breast cancer and positivity rates of each marker and panels.
