# Supplementary material for: Comparison of RNA Marker Panels for Circulating Tumor Cells and Evaluation of Their Prognostic Relevance in Breast Cancer
Source: Cancers (Basel). 2023 Feb 16;15(4):1271. doi: 10.3390/cancers15041271 (PMC9954525; doi:10.3390/cancers15041271)
Supplement: Supplementary file 1 [file cancers-15-01271-s001.zip › cancers-2221177-supplementary.pdf]

cancers

MDPI

Supplementary Material

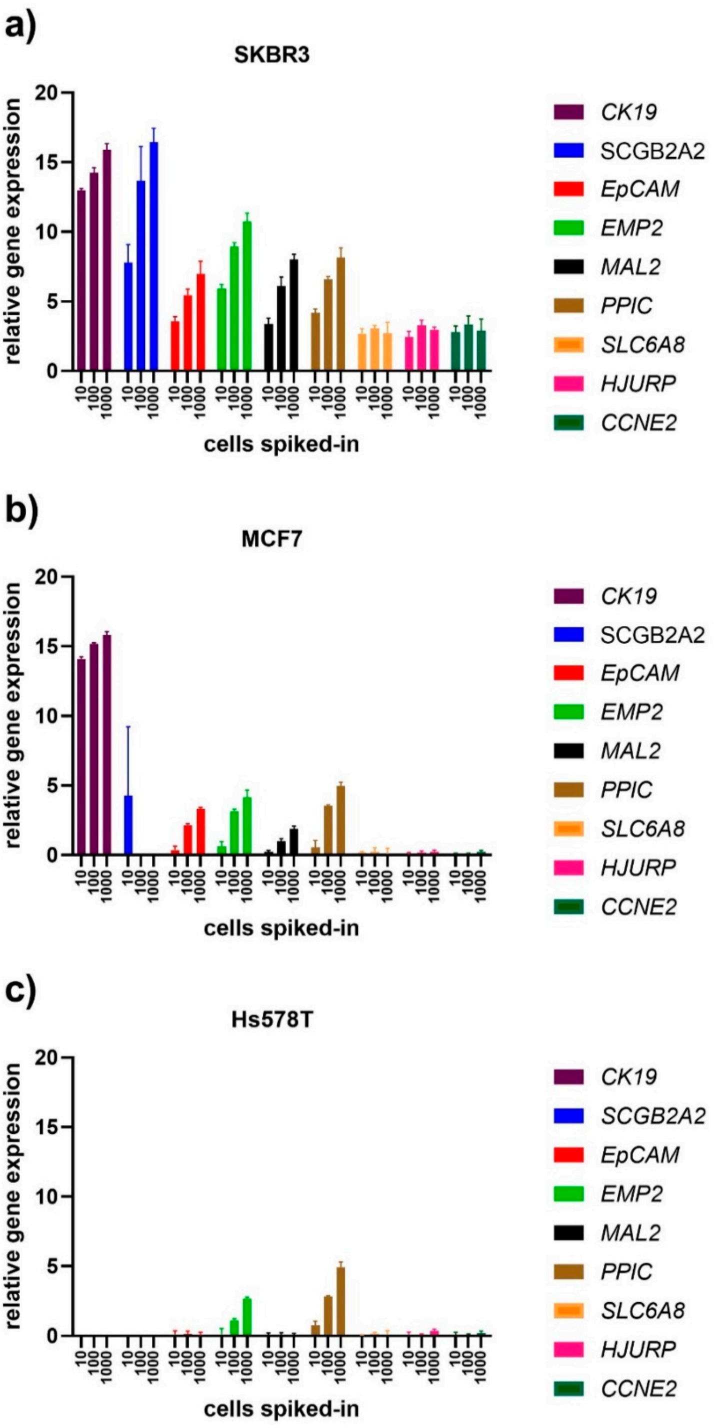

Figure S1. Results of spiking experiment with (a) SKBR3, (b) MCF7 and (c) Hs578T breast cancer cells and analysis of marker expression with qPCR. Relative gene expressions of CK19, SCGB2A2, EpCAM, EMP2, MAL2, PPIC, SLC6A8, HJURP and CCNE2 are shown in biological and technical replicates of 10, 100, 1000 tumor cells spiked into healthy donor blood. The bars depict the mean gene expression relative to an unspiked control sample, and the error bars the standard deviation.

Table S1. Characteristics of the 36 patients with metastatic breast cancer and positivity rates of each marker and panels**.** The associations of marker positivity and patients' characteristics were evaluated using a Fisher's exact test or a chi-square test where appropriate (*). Unadjusted p-values are shown. TNBC triple negative breast cancer.

	n	Panel 1	CK19	SCGB2A2	EPCAM	Panel 2	EMP2	SLC6A8	HJURP	MAL2	PPIC	CCNE2
**All patients**	36	-	-	-	-	-	-	-	-	-	-	-
**Mean age (range), years**	63.3 (30.3–82.6)	-	-	-	-	-	-	-	-	-	-	-
**Histopathological subtype**												
**luminal**	26	76,9%	23,1%	61,5 %	50,0 %	73,1 %	15,4 %	57,7 %	7,7 %	19,2 %	61,5 %	15,4 %
**HER2-positive**	4	50,0 %	0,0 %	50,0 %	25,0 %	75,0 %	0,0 %	0,0 %	0,0 %	0,0 %	75,0 %	0,0 %
**TNBC**	5	40,0 %	0,0 %	40,0 %	20,0 %	80,0 %	20,0 %	60,0 %	20,0 %	20,0 %	60,0 %	20,0 %
p		0.159	0.451	0.738	0.390	1.000	1.000	0.123	0.603	1.000	1.000	1.000
**Number of treatment lines received**												
**0**	8	75,0 %	12,5 %	75,0 %	12,5 %	62,5 %	0,0 %	37,5 %	0,0 %	12,5 %	50,0 %	12,5 %
**1**	12	58,3 %	8,3 %	58,3 %	33,3 %	66,7 %	16,7 %	58,3 %	16,7 %	8,3 %	58,3 %	16,7 %
**>1**	15	80,0 %	26,7 %	53,3 %	66,7 %	93,3 %	20,0 %	60,0 %	6,7 %	26,7 %	80,0 %	13,3 %
p		0.239	0.524	0.564	0.039	0.074	0.586	0.569	0.587	0.524	0.181	1.000
**Number of metastasis sites**												
**1**	18	72,2 %	11,1 %	55,6 %	50,0 %	77,8 %	11,1 %	55,6 %	5,6 %	16,7 %	55,6 %	11,1 %
**>1**	17	64,7 %	23,5 %	58,8 %	35,3 %	70,6 %	17,6 %	47,1 %	11,8 %	17,6 %	70,6 %	17,6 %
p		0.721*	0.391	0.709*	0.296*	0.706	0.650	0.739*	0.593	1.000	0.322*	0.650
**Bone metastasis**												
**yes**	30	66,7 %	20,0 %	60,0 %	36,7 %	70,0 %	13,3 %	50,0 %	6,7 %	13,3 %	60,0 %	10,0 %
**no**	5	80,0 %	0,0 %	40,0 %	80,0 %	100,0 %	20,0 %	60,0 %	20,0 %	40,0 %	80,0 %	40,0 %
p		1.000	0.561	0.631	0.141	0.297	0.561	0.528	0.380	0.195	0.630	0.139
**Liver metastasis**												
**yes**	10	80,0 %	30,0 %	60,0 %	50,0 %	70,0 %	20,0 %	60,0 %	20,0 %	30,0 %	60,0 %	20,0 %
**no**	25	64,0 %	12,0 %	56,0 %	40,0 %	76,0 %	12,0 %	48,0 %	4,0 %	12,0 %	64,0 %	12,0 %
p		0.447	0.322	1.000	0.712	0.694	0.610	0.711	0.190	0.322	1.000	0.610
**Lung metastasis**												
**yes**	12	58,3 %	8,3 %	50,0 %	33,3 %	75,0 %	8,3 %	25,0 %	8,3 %	8,3 %	75,0 %	16,7 %
**no**	23	73,9 %	21,7 %	60,9 %	47,8 %	73,9 %	17,4 %	65,2 %	8,7 %	21,7 %	56,5 %	13,0 %
p		0.451	0.640	0.721*	0.411*	1.000	0.640	0.035*	1.000	0.640	0.463	1.000
**Brain metastasis**												
**yes**	5	80,0 %	0,0 %	60,0 %	60,0 %	80,0 %	20,0 %	80,0 %	20,0 %	20,0 %	60,0 %	40,0 %
**no**	31	67,7 %	19,4 %	54,8 %	41,9 %	74,2 %	12,9 %	48,4 %	6,5 %	16,1 %	61,3 %	9,7 %
p		1.000	0.564	1.000	0.637	1.000	0.549	0.342	0.370	1.000	1.000	0.132

Table S2. Characteristics of the 90 patients with early breast cancer and positivity rates of each marker and panels. The associations of marker positivity and patients' characteristics were evaluated using a Fisher's exact test or a chi-square test where appropriate (*). Unadjusted p-values are shown. Bold p-values indicate significance after Bonferroni correction.

	n	Panel 1	CK19	SCGB2A2	EPCAM	Panel 2	EMP2	SLC6A8	HJURP	MAL2	PPIC	CCNE2
**All patients**	90	-	-	-	-	-	-	-	-	-	-	-
**Mean age (range), years**	62.2 (26.9 – 91.0)	-	-	-	-	-	-	-	-	-	-	-
**Estrogen receptor**												
**positive (score 3–8)**	71	22.5 %	12.7 %	8.5 %	8.5 %	25.4 %	8.5 %	16.9 %	4.2 %	2.8 %	5.6 %	9.9 %
**negative (score 0–2)**	13	7.7 %	0.0 %	7.7 %	0.0 %	15.4 %	7.7 %	7.7 %	0.0 %	0.0 %	0.0 %	7.7 %
p		0.451	0.343	1.000	0.584	0.724	1.000	0.681	1.000	1.000	1.000	1.000
**Progesterone receptor**												
**positive (score 3–8)**	63	19.0 %	11.1 %	7.9 %	7.9 %	25.4 %	9.5 %	17.5 %	3.2 %	3.2 %	6.3 %	9.5 %
**negative (score 0–2)**	21	23.8 %	9.5 %	9.5 %	4.8 %	19.0 %	4.8 %	9.5 %	4.8 %	0.0 %	0.0 %	9.5 %
p		0.755	1.000	1.000	1.000	0.769*	0.674	0.502	1.000	1.000	0.568	1.000
**HER2 (IHC)** [1]												
**positive**	19	10.5 %	5.3 %	5.3 %	0.0 %	26.3 %	10.5 %	15.8 %	0.0 %	0.0 %	5.3 %	5.3 %
**negative**	36	30.6 %	13.9 %	11.1 %	16.7 %	25.0 %	8.3 %	13.9 %	8.3 %	5.6 %	5.6 %	19.4 %
p		0.180	0.653	0.649	0.083	1.000	1.000	1.000	0.544	0.539	1.000	0.239
**Tumor grade**												
**1–2**	52	13.5 %	7.7 %	3.8 %	7.7 %	26.7 %	7.7 %	17.3 %	0.0 %	1.9 %	1.9 %	5.8 %
**3**	30	33.3 %	16.7 %	16.7 %	6.7 %	23.1 %	10.0 %	13.3 %	10.0 %	3.3 %	10.0 %	16.7 %
p		**0.047**	0.276	0.094	1.000	0.792*	0.703	0.760	0.046	1.000	0.136	0.135
**KI 67 label index**												
**<50** %	66	18.2 %	10.6 %	7.6 %	7.6 %	27.3 %	9.1 %	18.2 %	3.0 %	3.0 %	6.1 %	10.6 %
**≥50** %	17	29.4 %	11.8 %	11.8 %	5.9 %	11.8 %	5.9 %	5.9 %	5.9 %	0.0 %	0.0 %	5.9 %
p		0.303	1.000	0.793	1.000	0.485	1.000	0.486	0.610	1.000	0.696	1.000
**Lymphangioinvasio*n***												
**positive**	8	25.0 %	12.5 %	12.5 %	0.0 %	12.5 %	0.0 %	0.0 %	0.0 %	0.0 %	0.0 %	12.5 %
**negative**	75	20.0 %	10.7 %	8.0 %	8.0 %	25.3 %	9.3 %	17.3 %	4.0 %	2.7 %	5.3 %	9.3 %
p		0.664	1.000	0.522	1.000	0.673	1.000	0.346	1.000	1.000	1.000	0.572
**Relapse**												
**yes**	6	0.0 %	0.0 %	0.0 %	0.0 %	0.0 %	0.0 %	0.0 %	0.0 %	0.0 %	0.0 %	0.0 %
**no**	84	20.2 %	10.7 %	8.3 %	7.1 %	25.0 %	8.3 %	16.7 %	3.6 %	2.4 %	4.8 %	9.5 %
p		1.000	1.000	1.000	1.000	0.329	1.000	0.585	0.352	1.000	1.000	1.000

[1]ASCO CAP 2018 HER2 Testing for Breast Cancer Guidelines; Patients with equivocal HER2 status (n = 28) were excluded from the analysis.
